# Load-power relationship in older adults: The influence of maximal mean and peak power values and their associations with lower and upper-limb functional capacity

**DOI:** 10.3389/fphys.2022.1007772

**Published:** 2022-09-23

**Authors:** Diogo Luís Marques, Henrique Pereira Neiva, Daniel Almeida Marinho, Ivan Miguel Pires, Célia Nunes, Mário Cardoso Marques

**Affiliations:** ^1^ Department of Sport Sciences, University of Beira Interior, Covilhã, Portugal; ^2^ Research Center in Sports Sciences, Health Sciences and Human Development (CIDESD), Covilhã, Portugal; ^3^ Instituto de Telecomunicações, Universidade da Beira Interior, Covilhã, Portugal; ^4^ Department of Mathematics, University of Beira Interior, Covilhã, Portugal; ^5^ Centre of Mathematics and Applications, University of Beira Interior, Covilhã, Portugal

**Keywords:** muscle power, functional performance, medicine ball throw, chair stand, walking velocity, regression analysis, aging

## Abstract

Identifying the relative loads (%1RM) that maximize power output (P_max-load_) in resistance exercises can help design interventions to optimize muscle power in older adults. Moreover, examining the maximal mean power (MP_max_) and peak power (PP_max_) values (Watts) would allow an understanding of their differences and associations with functionality markers in older adults. Therefore, this research aimed to 1) analyze the load-mean and peak power relationships in the leg press and chest press in older adults, 2) examine the differences between mean P_max-load_ (MP_max-load_) and peak P_max-load_ (PP_max-load_) within resistance exercises, 3) identify the differences between resistance exercises in MP_max-load_ and PP_max-load_, and 4) explore the associations between MP_max_ and PP_max_ in the leg press and chest press with functional capacity indicators. Thirty-two older adults (79.3 ± 7.3 years) performed the following tests: medicine ball throw (MBT), five-repetition sit-to-stand (STS), 10-m walking (10 W), and a progressive loading test in the leg press and chest press. Quadratic regressions analyzed 1) the load-mean and peak power relationships and identified the MP_max-load_, MP_max_, PP_max-load_, and PP_max_ in both exercises, 2) the associations between MP_max_ and PP_max_ in the chest press with MBT, and 3) the associations between MP_max_ and PP_max_ in the leg press with STS_power_ and 10W_velocity_. In the leg press, the MP_max-load_ was ∼66% 1RM, and the PP_max-load_ was ∼62% 1RM, both for women and men (*p* > 0.05). In the chest press, the MP_max-load_ was ∼62% 1RM, and the PP_max-load_ was ∼56% 1RM, both for women and men (*p* > 0.05). There were differences between MP_max-load_ and PP_max-load_ within exercises (*p* < 0.01) and differences between exercises in MP_max-load_ and PP_max-load_ (*p* < 0.01). The MP_max_ and PP_max_ in the chest press explained ∼48% and ∼52% of the MBT-1 kg and MBT-3 kg variance, respectively. In the leg press, the MP_max_ and PP_max_ explained ∼59% of STS_power_ variance; however, both variables could not explain the 10W_velocity_ performance (*r* ^*2*^ ∼ 0.02). This study shows that the P_max-load_ is similar between sexes, is resistance exercise-specific, and varies within exercises depending on the mechanical power variable used in older adults. Furthermore, this research demonstrates the influence of the MBT as an upper-limb power marker in older adults.

## 1 Introduction

As people age, a sharp decrease in muscle power (i.e., the product of force and velocity) contributes to the loss of functional independence and increases the risk of falls and death in older adults (Reid and Fielding, 2012; Byrne et al., 2016; McKinnon et al., 2017). Therefore, measuring muscle power levels is essential for detecting early signs of mobility disability and designing preventive strategies, such as resistance training (Alcazar et al., 2018a; Beaudart et al., 2019). According to several studies, the spectrum of relative loads (% of one-repetition maximum [1RM]) that maximize power output (P_max-load_) in older people differs between resistance exercises (Potiaumpai et al., 2016; Strand et al., 2019). For example, the P_max-load_ range in the leg press is around 50%–70% 1RM, and in the chest press, between 40%–60% 1RM (de Vos et al., 2005; Potiaumpai et al., 2016; Ni and Signorile, 2017; Strand et al., 2019). Interestingly, a study that modeled the load-peak power relationship in participants aged ∼69 years did not observe differences between older women and men in the P_max-load_ in several resistance machines (Strand et al., 2019). According to the authors, the faster muscle power losses in older male adults than female counterparts might contribute to a convergence in muscle power production with age (Strand et al., 2019). Nevertheless, more research on older adults of similar or older ages is needed to corroborate or refute these observations.

Most studies with older people that modeled the load-power relationship in resistance exercises have primarily prioritized the analysis of the peak power variable (de Vos et al., 2005; Potiaumpai et al., 2016; Ni and Signorile, 2017). However, according to several authors, researchers should also consider mean power values when testing muscle power due to their measurement reliability and potential association with functional capacity in older adults (Alcazar et al., 2017; 2018a). Furthermore, it is essential to understand the differences between mechanical power variables when modeling the P_max-load_ for training prescription purposes. For example, regarding this matter, previous research with young trained adults observed that the P_max-load_ is exercise-specific and differs according to the mechanical power variable measured (Pallarés et al., 2014; Sánchez-Medina et al., 2014; Soriano et al., 2015, 2017; Martínez-Cava et al., 2019). These differences indicate that it is essential to define beforehand what mechanical power variable will be measured and monitored during the training program (considering the features of the linear encoder) to avoid erroneous decisions regarding training prescription. Nevertheless, to our knowledge, no known studies compared the differences in the P_max-load_ using the mean power (MP_max-load_) and peak power (PP_max-load_) values in lower and upper-limb resistance exercises in older people. Therefore, to improve the design of resistance training interventions, future research with older people must model the load-mean and peak power relationships in resistance exercises and examine eventual differences between MP_max-load_ and PP_max-load_ in the same exercise and the differences in MP_max-load_ and PP_max-load_ between resistance exercises.

In addition to analyzing the load-mean and peak power relationships to examine the pattern of mechanical power across a broad range of relative loads, it is also essential to examine the association between the maximal mean power (MP_max_) and peak power (PP_max_) values (Watts, W) with markers of functional capacity in older people. For example, several authors observed that the PP_max_ in the leg press and knee extension could explain 38% of the variance in the short physical performance battery test (i.e., balance, walk, and chair stand tests) in mobility-limited older adults aged 65 years or over (Bean et al., 2002). On the other hand, research with community-dwelling older people aged 70 years or over observed that leg press mean values could explain more of the short physical performance battery test variance than peak values (34% vs. 15%, respectively) (Alcazar et al., 2017). Nevertheless, research is scarce comparing the associations between MP_max_ and PP_max_ in the leg press with lower-limb functional capacity field tests, including chair stand and walking performance, meaning that this topic needs further investigation. Furthermore, to our knowledge, research is scarce regarding the associations between MP_max_ and PP_max_ in upper-limb resistance exercises, such as the chest press, with upper-limb functional capacity markers.

As suggested by some researchers, evaluating upper-limb muscle power can provide essential information regarding the functionality of older people due to its impact on performing the activities of daily living, such as standing up from a chair with the help of the arms and lifting and carrying groceries (Metter et al., 1997; Macaluso and De Vito, 2004; Candow and Chilibeck, 2005; Harris et al., 2011). In this matter, research with community-dwelling older adults aged ∼72 years found associations between the peak force applied during a modified push-up (knees on the ground) and the medicine ball throw (MBT) with 1.5 kg (*r* = 0.64) and 3 kg (*r* = 0.61) (Harris et al., 2011). Nevertheless, since the authors did not report the associations between MP_max_ and PP_max_ produced during the modified push-up with MBT, this analysis still needs to be conducted. In addition, selecting a resistance exercise performed in a seated position, such as the chest press, might be more representative of MBT performance than push-ups. Nevertheless, to our knowledge, no studies have yet assessed the association between MP_max_ and PP_max_ in the seated chest press with MBT performance in older people, representing a gap in the literature. Therefore, analyzing these relationships will allow an understanding of the applicability of the MBT as a functional field test to evaluate upper-limb muscle power in older adults.

Given the above considerations, the current research aimed to 1) analyze the load-mean and peak power relationships in the leg press and chest press in older women and men, 2) examine the differences between MP_max-load_ and PP_max-load_ within resistance exercises, 3) identify the differences between resistance exercises in MP_max-load_ and PP_max-load_, and 4) explore the associations between MP_max_ and PP_max_ in the leg press and chest press with functional capacity indicators. We hypothesized that the P_max-load_ in the leg press and chest press would be similar between older women and men (Strand et al., 2019). In addition, we hypothesized that the MP_max-load_ and PP_max-load_ would differ within and between resistance exercises (Pallarés et al., 2014; Sánchez-Medina et al., 2014; Martínez-Cava et al., 2019). Finally, we hypothesized that the MP_max_ and PP_max_ in the chest press would explain the MBT performance variance, while the MP_max_ and PP_max_ in the leg press would explain the performance variability in functional field tests for the lower limbs, including standing up from a chair and short-distance walking.

## 2 Materials and Methods

### 2.1 Study design

**FIGURE 1:**
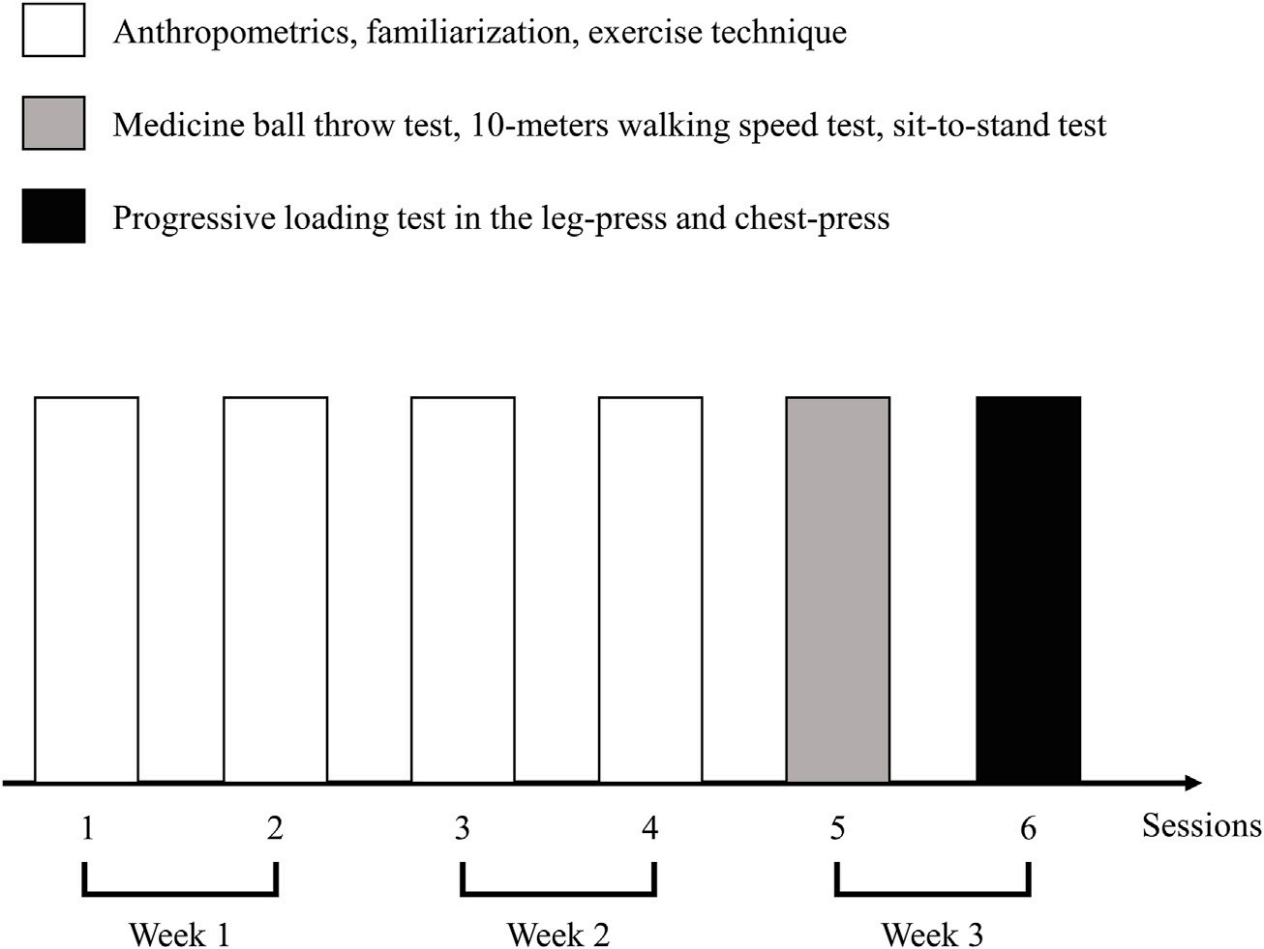
Illustration of the study design.

In this cross-sectional study, the participants went to a fitness health club for three consecutive weeks to perform two weekly sessions, separated by 48 h of rest. We dedicated the first 2 weeks to familiarization and anthropometric measures. During this period, we emphasized the proper execution technique of each exercise and movement velocity. Afterward, in the first session of the third week, the participants performed the following tests: MBT with 1 kg (MBT-1 kg) and 3 kg (MBT-3 kg), 10-m walking speed (10W), and five-repetition sit-to-stand (STS). After 48 h of rest, the participants performed a second session constituted by a progressive loading test in the leg press and chest press. An experienced researcher involved in the study and two certified senior fitness coaches supervised the procedures to guarantee safety and proper supervision during each exercise. In addition, verbal encouragement was provided during each exercise to motivate the participants to give a maximal effort. Figure 1 illustrates the study design.

### 2.2 Participants

We estimated a sample size of twenty-three participants to achieve a power of 80%, considering an alpha level of 0.05, two predictor variables (MP_max_ and PP_max_), and an *r* ^*2*^ of 0.38 based on the relationship between leg power and the short physical performance battery reported by Bean et al. (2002) (G*Power v3.1). Therefore, thirty-two older adults from residential care facilities and day centers were recruited to participate in this study (Table 1). We included male and female participants aged 65 years or more, able to walk and stand up from a chair independently, and willing to participate in the study. We excluded participants if they had physical dependency (Barthel Index score <60), cognitive decline (Mini-Mental State Examination [MMSE] cut-off scores: no years of schooling, <15 points; 1–11 years of school, <22 points; and >11 years of school, <27 points (Mendes et al., 2017)), musculoskeletal injuries in the previous 3 months, and terminal illness. The clinicians of the centers conducted the initial screening tests, including the Barthel Index and MMSE. According to the clinicians, all participants had no records of risk factors (e.g., uncontrolled hypertension and arrhythmia) that could prevent them from performing the exercises included in the study. Furthermore, all participants were classified as sedentary since they had no records of participating in regular physical exercise programs in the last 3 months. All participants were informed of the study procedures and signed written informed consent. The Ethical Committee of the University of Beira Interior approved this study (CE-UBI-Pj-2019-019).

**TABLE 1:** Participants’ characteristics.

Variable	Women (*n* = 17)	Men (*n* = 15)	Total (*n* = 32)
Age (years)	80.2 ± 7.8	78.3 ± 6.9	79.3 ± 7.3
Body mass (kg)	65.7 ± 10.2	75.0 ± 13.9	70.1 ± 12.8
Height (m)	1.49 ± 0.06	1.64 ± 0.08	1.56 ± 0.10
BMI (kg/m^2^)	29.5 ± 4.2	27.8 ± 4.6	28.7 ± 4.4
Barthel index score	90.6 ± 12.0	95.7 ± 9.8	93.0 ± 11.1
MMSE score	21.1 ± 3.8	24.1 ± 4.3	22.5 ± 4.3
10W_velocity_ (m·s^−1^)	1.6 ± 0.2	1.7 ± 0.4	1.6 ± 0.3
STS_power_ (W)	194.1 ± 53.6	259.7 ± 79.5	224.9 ± 73.8
MBT-1 kg (m)	3.1 ± 0.5	3.6 ± 0.9	3.3 ± 0.7
MBT-3 kg (m)	2.1 ± 0.3	2.6 ± 0.6	2.4 ± 0.5
1RM chest press (kg)	31.9 ± 6.4	44.4 ± 10.1	37.8 ± 10.4
1RM leg press (kg)	70.3 ± 14.7	87.5 ± 18.6	78.4 ± 18.6

Values are mean ± SD. Abbreviations: RM, repetition maximum; BMI, body mass index; MBT, medicine ball throw; MMSE, mini-mental state examination; STS, five-repetition sit-to-stand; 10W, 10-m walking.

### 2.3 Measurements

#### 2.3.1 Seated medicine ball throw

The participants held the ball on their chest and threw it as far as possible while seated on a chair (0.49 m) (Marques et al., 2020). They performed three trials with 1 and 3 kg balls, interspersed with 1-min rest. We measured the distance (m) from the chest to where the balls landed using a tape measure and analyzed the best attempts.

#### 2.3.2 Ten-meters walking speed

The participants walked 10-m linearly at the maximal intended velocity on an indoor wooden track (Pereira et al., 2012). They performed three trials, separated by 3 min of rest. We measured the time (s) using photoelectric cells (Race Time Kit 2, Microgate, Italy) and estimated the mean velocity (10-m divided by time; 10W_velocity_, in m·s^−1^) of the best trial.

#### 2.3.3 Five-repetition sit-to-stand

The participants stood up and sat down on a chair (0.49 m) with their arms crossed over the chest five times (Alcazar et al., 2018b). They performed two trials, separated by 2-min rest. We measured the time (s) using a stopwatch (Casio HS-3V-1R, Japan) and estimated the STS mean power (STS_power_, in W) using a validated equation (Alcazar et al., 2018a), and selected the best attempt.

#### 2.3.4 Progressive loading test in the leg press and chest press

In the leg press (Leg press G3, Matrix, United States), the participants were seated on the bench with their hands on the side handles. They placed their feet on the platform shoulder-width apart, knees at 90°, and back in contact with the seat. In the chest press (Chest press G3, Matrix, United States), the participants were seated on the bench with the handgrips at mid-chest, shoulders abducted, elbows flexed at 90°, and handles grabbed with a full grip. The leg press warm-up consisted of seven repetitions with 20.5 kg plus five repetitions with 29.5 kg, while the chest press warm-up consisted of seven repetitions with 5.7 kg plus five repetitions with 10.2 kg. The initial weight was 29.5 and 10.2 kg in the leg press and chest press, respectively. We increased the weight by 10 kg in the leg press and 5 kg in the chest press until the participants achieved the 1RM. If they could not perform one correct repetition, we decreased the weight by 1–5 kg. The participants performed the repetitions at the maximal intended velocity, and we asked them to perform three repetitions whenever possible to guarantee proper data collection. The inter-set rest was 3 min for three repetitions and 5 min for two repetitions (Marques et al., 2021). Using the procedures described elsewhere (Marques et al., 2020), we coupled a linear velocity transducer (T-Force System, Ergotech, Spain) to the leg press and chest press machines to calculate each repetition’s mean and peak power. We selected the maximal mean and peak power values attained with each weight for analysis. The set’s average number was 6.4 ± 1.7 and 6.7 ± 1.5 in the leg press and chest press, respectively.

### 2.4 Statistical analysis

We examined the assumption of normality of the data using the Kolmogorov-Smirnov test. We used standard statistical methods to calculate means, standard deviations (SD), 95% confidence intervals (CI), Pearson correlation coefficients (*r*), the adjusted coefficient of determination (*r* ^2^), and the standard error of the estimate (SEE). Quadratic regressions analyzed 1) the load-mean and peak power relationships in the leg press and chest press and identified the MP_max-load_ (% 1RM), MP_max_ (W), PP_max-load_ (% 1RM), and PP_max_ (W) in the leg press and chest press in women and men, 2) the associations between MP_max_ and PP_max_ in the chest press with MBT-1 kg and MBT-3 kg, and 3) the associations between MP_max_ and PP_max_ in the leg press with 10W_velocity_ and STS_power_. We used quadratic regressions to analyze the associations between MP_max_ and PP_max_ with functional capacity markers due to the curvilinear relationship between muscle power and functional capacity (Bean et al., 2002; Cuoco et al., 2004; Marsh et al., 2006; Byrne et al., 2016). Independent samples *t*-test analyzed 1) the differences between sexes in absolute mean and peak power values (W) in the leg press and chest press for each relative load, including the MP_max-load_ and PP_max-load_, and 2) the differences between sexes in MP_max-load_ and PP_max-load_ in the leg press and chest press. A repeated-measures ANOVA with post hoc Bonferroni tests analyzed the differences between MP_max_/PP_max_ in the leg press and chest press with absolute power values (W) at different relative loads in men and women. Paired samples *t*-test analyzed 1) the differences between MP_max-load_ and PP_max-load_ within resistance exercises, and 2) the differences between resistance exercises in MP_max-load_ and PP_max-load_. We performed the statistical analyses in Microsoft Office Excel^®^ (Microsoft Inc., Redmond, WA, United States) and SPSS v27 (SPSS Inc., United States) and set the significance level at *p* < 0.05. We designed the figures in GraphPad Prism v7 (GraphPad Inc., San Diego, CA, United States).

## 3 Results

### 3.1 Load-mean and peak power relationships in the leg press in women and men

**FIGURE 2:**
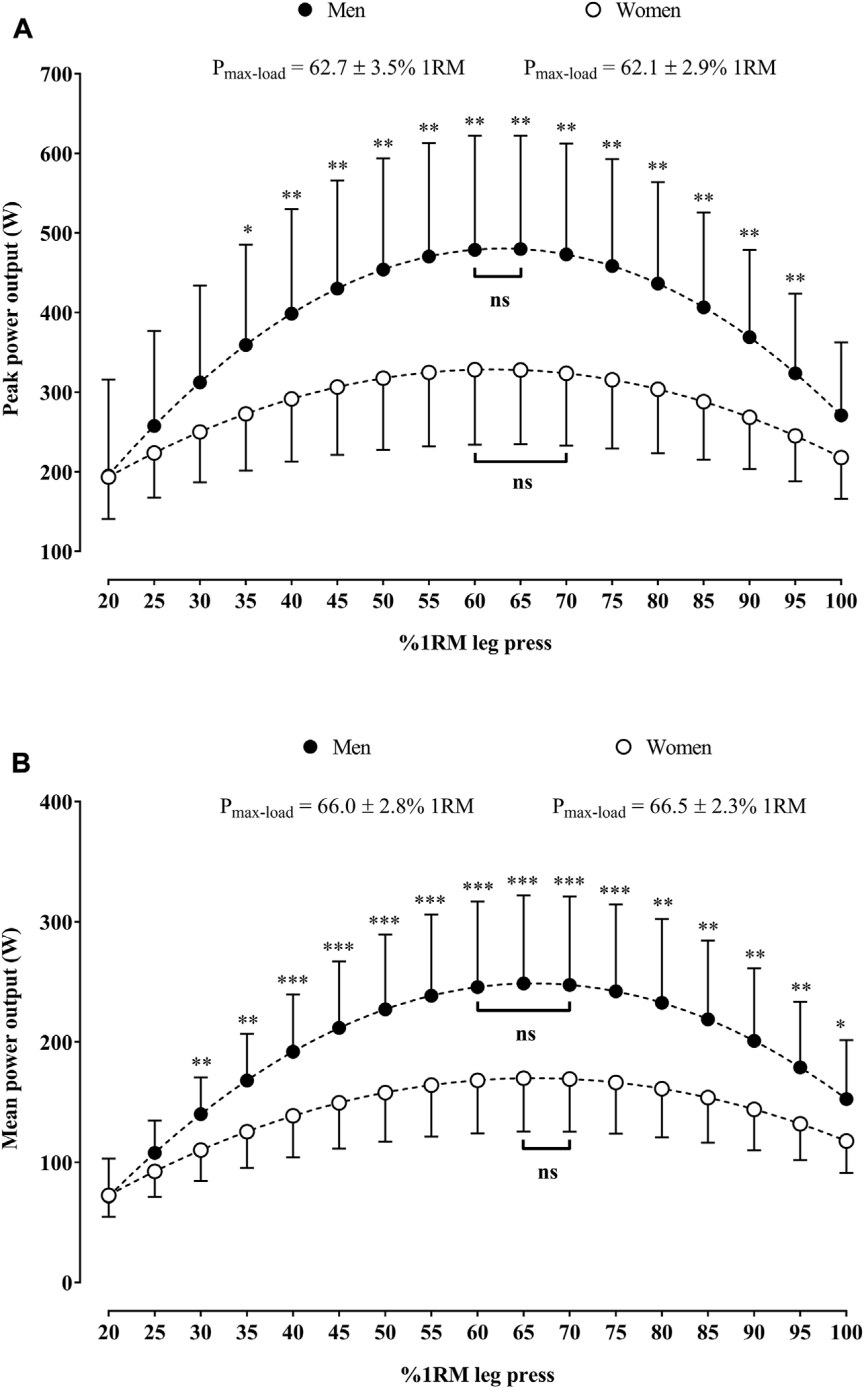
Load-peak **(A)** and mean power **(B)** relationships in the leg press for older women and men. *p < 0.05, **p < 0.01, and ***p < 0.001 indicate significant differences between sexes in the absolute mean or peak power against the same relative load. Square brackets indicate the range of relative loads at which the power output was not statistically different (ns) than the P_max-load_. Abbreviation: P_max-load_, relative load that maximizes the power output; RM, repetition maximum.

Figure 2 shows the load-mean and peak power relationships in the leg press in older women and men. Men presented higher absolute peak power values than women at 35%–95% 1RM (Figure 2A) and higher absolute mean power values at 30%–100% 1RM (Figure 2B). The PP_max-load_ in the leg press did not differ between men and women (*p* = 0.59). In men, the PP_max_ was not different from peak power values associated with loads at 60%–65% 1RM (*p* > 0.05), while in women, the PP_max_ was not different from peak power values associated with loads at 60%–70% 1RM (*p* > 0.05) (Figure 2A). The MP_max-load_ in the leg press did not differ between men and women (*p* = 0.62). In men, the MP_max_ was not different from mean power values associated with loads at 60%–70% 1RM (*p* > 0.05), while in women, the MP_max_ was not different from mean power values associated with loads at 65%–70% 1RM (*p* > 0.05) (Figure 2B).

### 3.2 Load-mean and peak power relationships in the chest press in women and men

**FIGURE 3:**
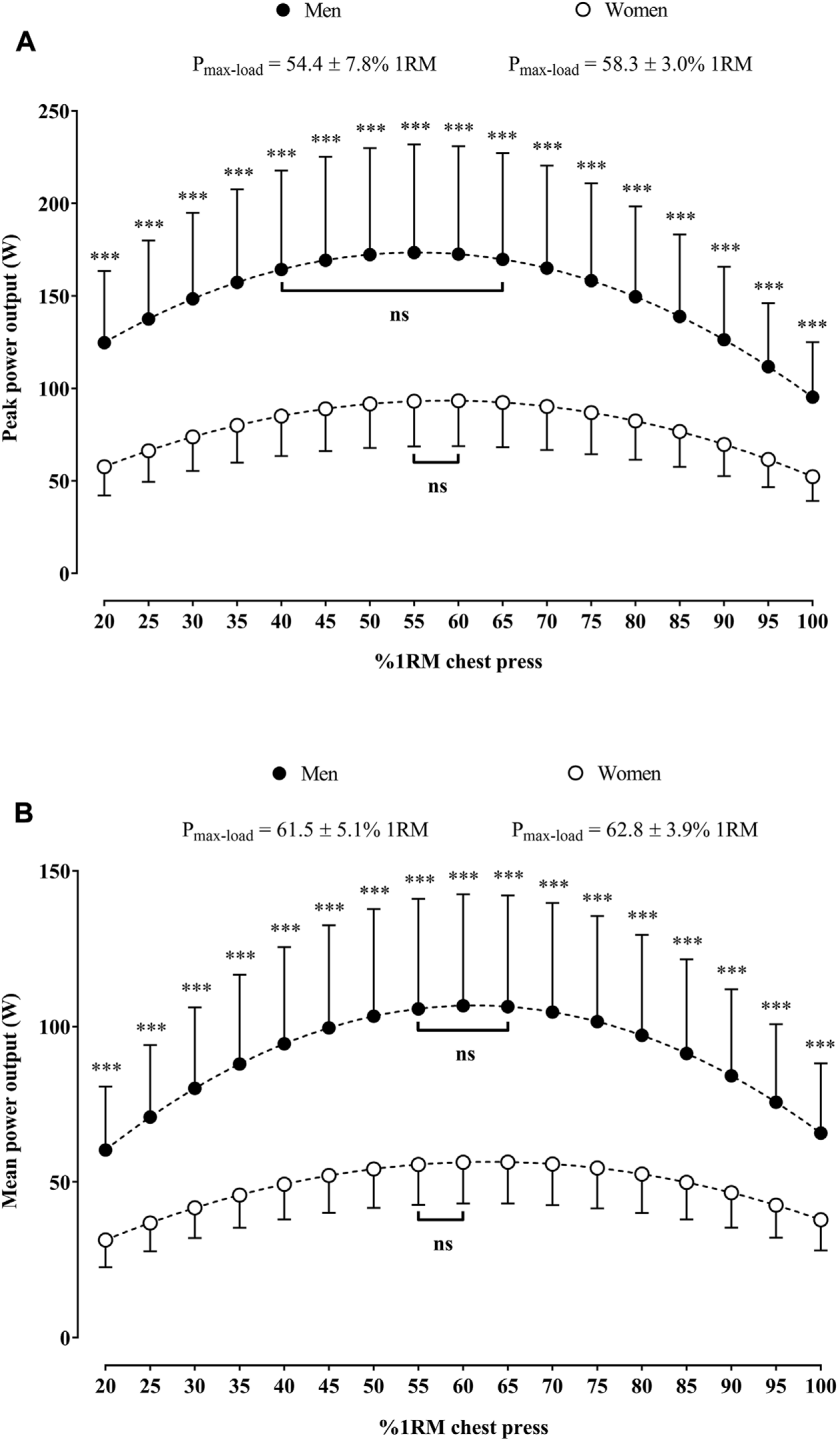
Load-peak **(A)** and mean power **(B)** relationships in the chest press for older women and men. *p < 0.05, **p < 0.01, and ***p < 0.001 indicate significant differences between sexes in the absolute mean or peak power against the same relative load. Square brackets indicate the range of relative loads at which the power output was not statistically different (ns) than the P_max-load_. Abbreviation: P_max-load_, relative load that maximizes the power output; RM, repetition maximum.

Figure 3 shows the load-mean and peak power relationships in the chest press in older women and men. Men presented higher absolute peak and mean power values than women at 20%–100% 1RM (Figures 3A,B, respectively). The PP_max-load_ in the chest press did not differ between men and women (*p* = 0.09). In men, the PP_max_ was not different from peak power values associated with loads at 40%–65% 1RM (*p* > 0.05), while in women, the PP_max_ was not different from peak power values associated with loads at 55%–60% 1RM (*p* > 0.05) (Figure 3A). The MP_max-load_ in the chest press did not differ between men and women (*p* = 0.41). In men, the MP_max_ was not different from mean power values associated with loads at 55%–65% 1RM (*p* > 0.05), while in women, the MP_max_ was not different from mean power values associated with loads at 55%–60% 1RM (*p* > 0.05) (Figure 3B).

### 3.3 Differences between leg press vs. chest press in mean P_max-load_ and peak P_max-load_

Table 2 shows differences between the leg press vs. chest press in PP_max-load_ for men and women (*p* < 0.01). In addition, there were differences between the leg press vs. chest press in MP_max-load_ for men and women (*p* < 0.01).

**TABLE 2:** Differences between leg press vs. chest press using the peak P_max-load_ and mean P_max-load_ in both sexes.

		Leg press		Chest press		
Sex	Variable	Mean ± SD	95% CI	Mean ± SD	95% CI	*p*-value*
Male	Peak P_max-load_ (% 1RM)	62.7 ± 3.5	60.9–64.4	54.4 ± 7.8	50.5–58.3	0.004
Female	Peak P_max-load_ (% 1RM)	62.1 ± 2.9	60.7–63.4	58.3 ± 3.0	56.9–59.7	<0.001
Male	Mean P_max-load_ (% 1RM)	66.0 ± 2.8	64.6–67.4	61.5 ± 5.1	58.9–64.1	0.004
Female	Mean P_max-load_ (% 1RM)	66.5 ± 2.3	65.4–67.6	62.8 ± 3.9	61.0–64.7	0.009

Values are mean ± standard deviation (SD) with 95% confidence intervals (CI). ^*^ Paired samples *t*-test. Abbreviations: P_max-load_, relative load that maximizes power output; RM, repetition maximum.

### 3.4 Differences between mean P_max-load_ vs. peak P_max-load_ within resistance exercises

Table 3 shows differences between PP_max-load_ vs. MP_max-load_ in the leg press for men and women (*p* < 0.01). In addition, there were differences between PP_max-load_ vs. MP_max-load_ in the chest press for men and women (*p* < 0.001).

**TABLE 3:** Differences between peak P_max-load_ vs. mean P_max-load_ in the leg press and chest press in both sexes.

		Peak P_max-load_		Mean P_max-load_		
Sex	Variable	Mean ± SD	95% CI	Mean ± SD	95% CI	*p*-value*
Male	Leg press (% 1RM)	62.7 ± 3.5	60.9–64.4	66.0 ± 2.8	64.6–67.4	0.004
Female	Leg press (% 1RM)	62.1 ± 2.9	60.7–63.4	66.5 ± 2.3	65.4–67.6	<0.001
Male	Chest press (% 1RM)	54.4 ± 7.8	50.5–58.3	61.5 ± 5.1	58.9–64.1	<0.001
Female	Chest press (% 1RM)	58.3 ± 3.0	56.9–59.7	62.8 ± 3.9	61.0–64.7	<0.001

Values are mean ± standard deviation (SD) with 95% confidence intervals (CI). ^*^ Paired samples *t*-test. Abbreviations: P_max-load_, relative load that maximizes power output; RM, repetition maximum.

### 3.5 Associations between maximal mean power and peak power in the leg press and chest press with functional capacity markers

**FIGURE 4:**
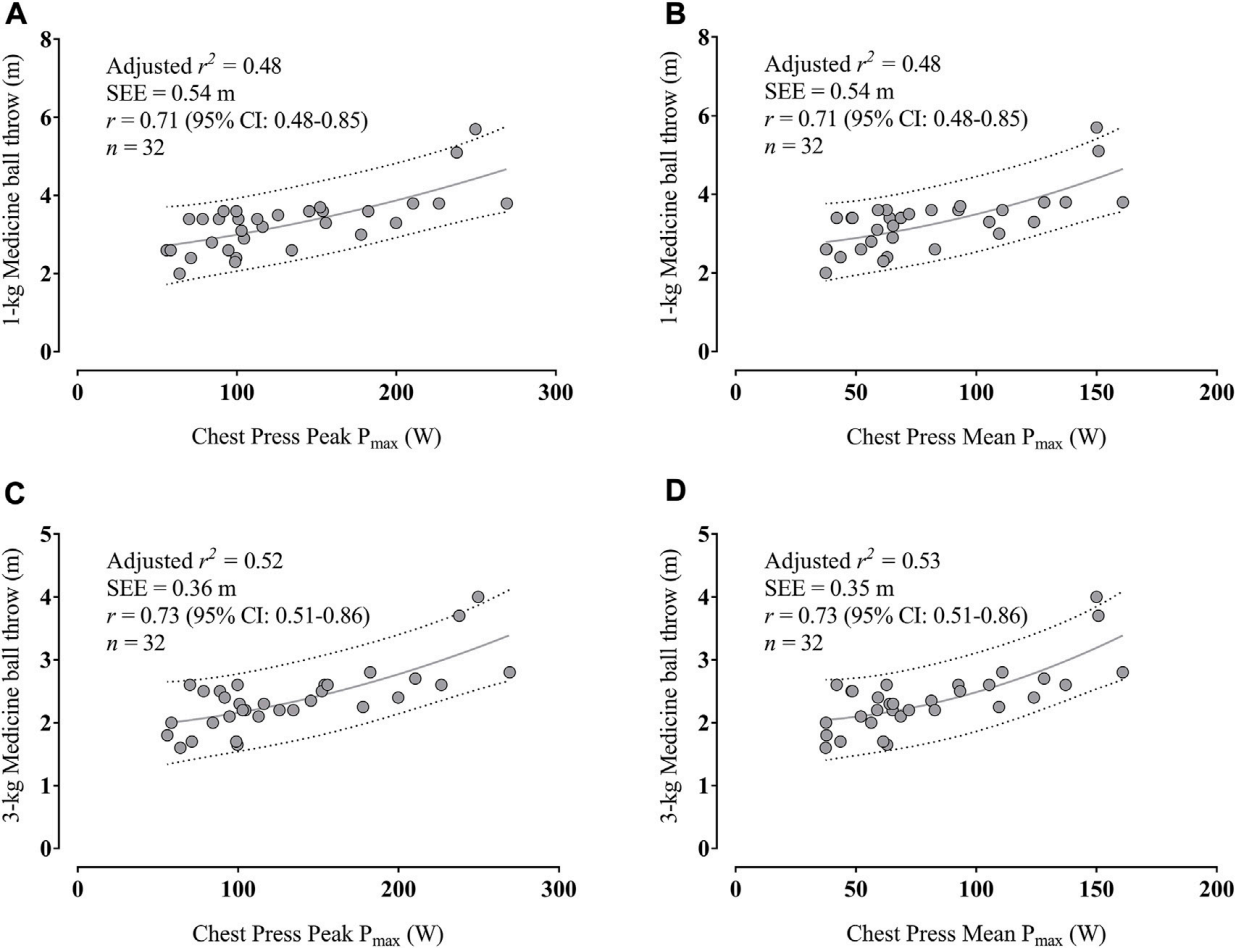
Associations between maximal peak power **(A)** and mean power output **(B)** in the chest press with 1-kg medicine ball throw and between peak power **(C)** and mean power output **(D)** with 3-kg medicine ball throw; Dotted lines indicate the prediction intervals. Abbreviation: CI, confidence interval; P_max_, maximal power output; SEE, standard error of the estimate.

Figure 4A indicates that the PP_max_ in the chest press explained 48% of MBT-1 kg variance, while Figure 4B shows that the MP_max_ in the chest press explained 48% of MBT-1 kg variance. In addition, Figure 4C reveals that the PP_max_ in the chest press explained 52% of MBT-3 kg variance, while Figure 4D shows that the MP_max_ in the chest press explained 53% of MBT-3 kg variance.

**FIGURE 5:**
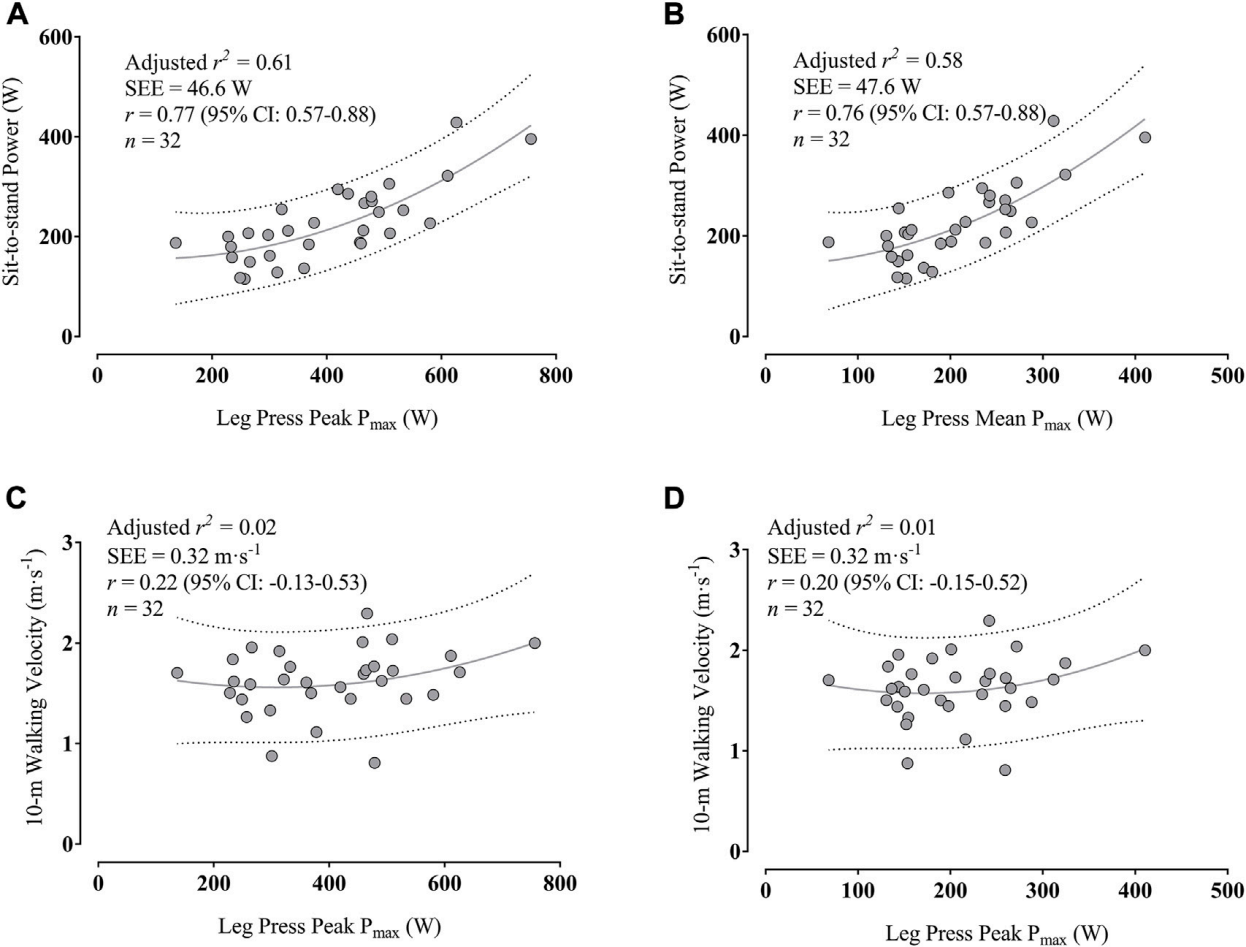
Associations between maximal peak power **(A)** and mean power output **(B)** in the leg press with sit-to-stand power and between peak power **(C)** and mean power output **(D)** with 10-m walking velocity; Dotted lines indicate the prediction intervals. Abbreviation: CI, confidence interval; P_max_, maximal power output; SEE, standard error of the estimate.

Figure 5A indicates that the PP_max_ in the leg press explained 61% of STS_power_ variance, while Figure 5B shows that the MP_max_ in the leg press explained 58% of STS_power_ variance. In addition, Figure 5C reveals that the PP_max_ in the leg press only explained 2% of 10W_velocity_ variance, while Figure 5D shows that the MP_max_ in the leg press only explained 1% of 10W_velocity_ variance.

## 4 Discussion

### 4.1 Main findings

The current study aimed to 1) analyze the load-mean and peak power relationships in the leg press and chest press in older women and men, 2) examine the differences between MP_max-load_ and PP_max-load_ within resistance exercises, 3) identify the differences between resistance exercises in MP_max-load_ and PP_max-load_, and 4) explore the associations between MP_max_ and PP_max_ in the leg press and chest press with functional capacity indicators. The main findings of the current study were: 1) the MP_max-load_ and PP_max-load_ in the leg press and chest press are similar between older women and men, 2) the MP_max-load_ and PP_max-load_ differ between resistance exercises, meaning that they are exercise-specific, 3) the P_max-load_ varies in the same resistance exercise depending on the mechanical power variable chosen to measure, 4) the MP_max_ and PP_max_ in the chest press similarly explain the variability in MBT-1 kg and MBT-3 kg performance, and 5) the MP_max_ and PP_max_ in the leg press similarly explain the STS_power_ variance; however both mechanical variables could not explain the variability in 10W_velocity_ performance.

### 4.2 Load-mean and peak power relationships in the leg press and chest press in older women and men

The results of this study showed that the MP_max-load_ and PP_max-load_ in the leg press and chest press did not differ between older women and men, which agrees with previous findings, particularly for the peak power values (Strand et al., 2019). This convergence in muscle power production between sexes might be related to the more significant and faster age-related losses of muscle power in men than women during aging (Edwén et al., 2014; Strand et al., 2019). The results also showed that the load-power relationship in older adults is resistance exercise-specific, thus corroborating the results of previous observations (Strand et al., 2019). For example, the PP_max-load_ in the leg press and chest press was around 60% and 55% 1RM, respectively, which agrees with previous findings (de Vos et al., 2008; Potiaumpai et al., 2016; Strand et al., 2019). On the other hand, the MP_max-load_ in the leg press and chest press was unknown until the completion of our study. Compared to PP_max-load,_ the MP_max-load_ in the leg press and chest press increased to around 66% and 62% 1RM, respectively. Despite its novelty in older populations, these data also indicate that the P_max-load_ differs between mechanical power variables in older adults, as observed in young adults (Pallarés et al., 2014; Sánchez-Medina et al., 2014; Martínez-Cava et al., 2019). Although most studies with older adults analyzed the P_max-load_ using the peak power variable (de Vos et al., 2005; Potiaumpai et al., 2016; Ni and Signorile, 2017; Strand et al., 2019), several authors observed higher reliability using mean values than peak values when conducting a progressive loading test in the leg press with this population (Alcazar et al., 2017). However, since no study had yet presented data concerning the MP_max-load_ in resistance exercises, these results present preliminary evidence for clinicians and researchers who want to collect mean power values to estimate the P_max-load_. In addition, these results also alert the importance of defining the mechanical power variable beforehand to be monitored during the intervention to avoid misinterpreting information during its course.

The current research also demonstrated that the P_max-load_ range in the leg press (∼60%–70% 1RM) and chest press (∼40%–65% 1RM) was narrower than those observed for younger populations when using, for example, the squat or bench press exercises (∼30%–70% 1RM) (Soriano et al., 2015; Soriano et al.,2017). These differences might be attributed to the progressive reduction in size and number of fast-twitch muscle fibers in the lower and upper limbs with aging, which negatively affects the elbow and knee extensor’s power capacity (Metter et al., 1997; Candow and Chilibeck, 2005; Korff et al., 2014). In addition, as observed in our data, the P_max-load_ range in the leg press was narrower than the chest press, which might be associated with the higher muscle power production losses in the lower limbs than in the upper limbs during aging (Macaluso and De Vito, 2004; Candow and Chilibeck, 2005). According to the literature, a significant reduction in physical activity with age and greater use of the upper limbs than the lower limbs to perform the activities of daily living (e.g., using arms to help to stand up from a chair) might contribute to higher decreases in lower limb’s power than upper limb’s power (Macaluso and De Vito, 2004; Candow and Chilibeck, 2005). Therefore, these results suggest a broad spectrum of relative loads to maximize the upper-limb muscle power and a narrow range of relative loads to maximize the lower-limb muscle power in older adults. Nevertheless, future research should analyze if training only with the P_max-load_ improves older adults’ muscle power to a greater extent than a broader range of relative loads.

### 4.3 Associations between maximal mean and peak power values in the leg press and chest press with functional capacity markers

The regression analysis showed that the MP_max_ and PP_max_ in the chest press could similarly explain the MBT-1 kg and MBT-3 kg performance. These data reinforce the influence of the MBT as an indicator of muscle power and functionality in older adults (Harris et al., 2011). Furthermore, although the relationship between the chest press power and functional capacity in older adults is scarce, earlier findings demonstrated a correlation between the chest press peak power and self-reported functional status (lower scores representing better functional status) (*r* = −0.35) in older women (Foldvari et al., 2000). Consequently, considering the associations between chest press muscle power with MBT, it can be suggested that the MBT seems an essential indicator of the capacity to perform the activities of daily living independently in older adults, such as lifting and carrying groceries and boxes, opening jars, rising from a chair with the help of the arms, and even catching oneself to prevent a fall (Adams et al., 2001; Candow and Chilibeck, 2005; Harris et al., 2011). Based on this information, clinicians, sport-related professionals, and researchers can administer the MBT test to analyze the upper-limb muscle power capacity and derive information regarding the functional ability of older adults.

As for the regression analysis in the lower limbs, the MP_max_ and PP_max_ in the leg press could similarly explain the variability in the STS_power_ performance. These results reinforce the substantial impact of lower-limb muscle power on explaining the variability during sit-to-stand transitions in older adults (Byrne et al., 2016). However, neither MP_max_ nor PP_max_ in the leg press could explain the variance in 10W_velocity_ performance. These results were surprising and unexpected since previous research found that leg press power could explain the variance in walking speed performance in older adults (Bean et al., 2002; Puthoff and Nielsen, 2007). Nevertheless, it is essential to note that the latter investigations that assessed the relationships between leg power and walking performance were conducted with mobility-limited older adults, unlike our study. Therefore, the impaired physical condition might have influenced the relationship between lower-limb muscle power with walking performance. Interestingly, research with community-dwelling older adults with similar maximal walking velocity values as our participants (1.6–2.0 m s^−1^) found that hip and ankle muscle strength were better predictors of maximal walking speed than leg strength (Uematsu et al., 2014; Muehlbauer et al., 2018). Therefore, it is essential to consider that hip and ankle strength might better account for the variance in walking performance than leg strength in older adults without mobility impairments (Muehlbauer et al., 2018). Nevertheless, future large-scale research is necessary to determine the influence of leg, hip, and ankle power and strength on maximal walking performance in older adults with and without mobility limitations.

Of note, the range of *r* ^*2*^ values observed in our study is in line with previous research (Byrne et al., 2016), which indicates that a large part of the variance in functional capacity is to be explained by other outcomes (Puthoff and Nielsen, 2007). For example, aerobic endurance, balance, flexibility, agility, and even the fear of falling might explain the variance in functional capacity in older adults (Puthoff and Nielsen, 2007). Therefore, future research should examine, along with lower and upper-limb muscle power, what physiological and psychological indices play a significant role in explaining the variability in functional capacity in older adults.

### 4.4 Study limitations and future research

The current study presents several limitations that we need to address. Firstly, a cross-sectional design does not allow us to establish causal relationships between muscle power with functional capacity in the tested population. In this perspective, future longitudinal studies with older adults should examine the effects of resistance training on muscle power and functional capacity and determine their relationships to support causal links. Secondly, although the sample size calculation determined that twenty-three participants were needed to obtain a statistical power of 80%, the actual number of participants is insufficient to generalize the results to other older adults. In addition, considering that our participants were functionally independent, caution should be taken when generalizing these results to mobility-limited older adults. Finally, including physiological and psychological outcomes would be helpful to examine if, along with lower and upper-limb muscle power measures, they could increase the capacity to explain the remaining part of the variance in functional capacity in older adults. Therefore, future research should consider the limitations mentioned above and conduct large-scale, longitudinal, and experimental studies to examine the physiological and psychological mechanisms that better explain the variability in functional capacity in older adults.

## 5 Conclusion

This study showed that the P_max-load_ in the leg press and chest press are similar between older women and men. Nevertheless, the P_max-load_ is exercise-specific and varies according to the mechanical power variable chosen for analysis. Therefore, from an applied perspective, this information can be helpful for clinicians, sport-related professionals, and researchers to design experimental interventions oriented to optimizing lower and upper-limb muscle power and functional capacity in older adults. In addition, the current research demonstrated the influence of the MBT exercise as a functional capacity field test for assessing upper-limb muscle power in older adults.

## Data Availability

The raw data supporting the conclusion of this article will be made available by the authors, without undue reservation.
